# A Case of Drug-Induced Liver Injury Secondary to Natalizumab

**DOI:** 10.1155/2020/7976585

**Published:** 2020-06-16

**Authors:** Phillip P. Santoiemma, Haripriya Maddur, Christopher M. Moore

**Affiliations:** ^1^Department of Internal Medicine, Northwestern University Feinberg School of Medicine, Chicago, IL, USA; ^2^Department of Gastroenterology and Hepatology, Northwestern University Feinberg School of Medicine, Chicago, IL, USA

## Abstract

A 33-year-old Caucasian female with relapsing-remitting multiple sclerosis presented with abdominal pain, nausea, and vomiting and was found to have acute liver injury. After thorough investigation, she was diagnosed with drug-induced liver injury (DILI) thought secondary to redosing of Natalizumab therapy.

## 1. Introduction

Drug-induced liver injury (DILI) can arise following the use of various medications, with a rather wide presentation: (1) hepatocellular, cholestatic, or mixed liver injury pattern; (2) in the setting of acute or chronic use; and (3) intrinsically or idiosyncratically in relation to dosing [1]. Direct hepatotoxicity is the most common form of DILI and injury to the liver is often intrinsic and related to dose response [1]. Indirect or idiosyncratic hepatotoxicity is less predictable and unrelated to dosing and timing of medication and often difficult to diagnose. Overall, the incidence of DILI annually is around 0.1–0.01%, and it is the most common cause of acute liver failure in the United States [1, 2]. We present a rare case of DILI secondary to redosing of Natalizumab.

## 2. Case Report

A 33-year-old Caucasian female with relapsing-remitting multiple sclerosis (RRMS) of 5 years duration presented with worsening fatigue, nausea and vomiting, and sharp right upper quadrant abdominal pain that began the morning of presentation. She denied any recent alcohol abuse, sick contacts, or recent travel. She denied the use of new medications or herbal agents. Historically, her RRMS was treated with interferon beta-1a medications but switched to Natalizumab after one year due to disease progression. She had subsequently been maintained on Natalizumab for 3 years. However, due to an attempt at pregnancy, she was briefly switched to therapy with dimethyl fumarate. During her nine-month course of the new therapy, she once again had symptom progression and was reinitiated on Natalizumab one day before presentation; of note, she never became pregnant.

Her examination revealed a tender abdomen, primarily in the right upper quadrant, but was otherwise unremarkable including a normal mental status. Laboratory evaluation revealed normal electrolytes, renal function, hemoglobin of 16 g/dl, platelets of 209,000 per *μ*L, and white blood cell count of 7,100 *μ*L. Her hepatic panel revealed an alanine aminotransferase (ALT) of 3,855 U/L, aspartate aminotransferase (AST) 932 U/L, total bilirubin 2.8 mg/dL, alkaline phosphatase 70 U/L, total protein 6.6 g/dL, and albumin 4.4 g/dL and INR 1.3; prior liver function tests were within normal limits drawn a 10 days before admission as well as during the prior 3-year period she was on Natalizumab. Her urine drug screen, acetaminophen, salicylate, and alcohol levels were negative and her urine HcG was negative. Viral hepatitis serologies were negative including hepatitis A, B, and C serologies as well as CMV, EBV, and HSV viral loads. Additional testing for anti-mitochondrial antibody, anti-smooth muscle antibody, and anti-nuclear antibody was negative.

**Figure 1:**
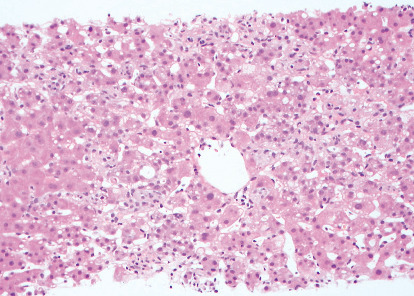
Liver biopsy (DPAS stain) demonstrating clusters of sinusoidal foamy histiocytes consistent with resolving hepatitis. Additional histology performed with trichrome, reticulin, and iron stains was unremarkable.

A liver ultrasound demonstrated patent vasculature of the liver with normal resistive indices in the hepatic arteries and with no sonographic abnormalities of the liver or biliary tree. A percutaneous ultrasound-guided liver biopsy revealed pathology consistent with resolving hepatitis, specifically presence of foamy histiocytes without overt necrosis (Figure 1).

Her aminotransferases subsequently improved with supportive care including intravenous fluids and frequent monitoring after 24 hours, specifically ALT decreased from 3,855 U/L to 1,320 U/L, AST decreased from 932 U/L to 88 U/L, and bilirubin decreased from 2.8 mg/dl to 1.8 mg/dL. Additionally, her abdominal pain spontaneously resolved during hospitalization and she was discharged home. Unfortunately, the patient was lost to follow-up to our health system.

## 3. Discussion

Natalizumab is a humanized monoclonal antibody against alpha4-integrin, which participates in cell adhesion and is used in the treatment of RRMS as well as Crohn's disease [3]. Natalizumab is thought to reduce the migration of T-cells that cross into the blood-brain barrier or small intestinal venular endothelium and thus reduce T-cell homing and subsequent inflammation [3]. The primary side effects include headache, fatigue, and infection and rarely progressive multifocal leukoencephalopathy. Even rarer are reports of Natalizumab-induced hepatotoxicity.

Overall, Natalizumab is a safe and very effective drug for RRMS with a rate of serious adverse events of 8% and elevated liver function tests in just 0.1% of patients in an ongoing, prospective multinational study [4]. It is reported that <5% of patients will have mild aminotransferase elevations on therapy, but <1% of patients will progress to fulminant liver failure [5]. In a recent review of the literature, Natalizumab has been associated with acute liver injury and drug-induced autoimmune hepatitis, but not pure acute liver failure [6]. Interestingly, most of the reports of acute liver injury are not after the first drug infusion, but rather upon subsequent dosing. The liver injury pattern is also spectral, i.e., hepatocellular through cholestatic; this particular case presented with hepatocellular injury.

Although in most cases the cause of DILI is difficult to assess [7], in this particular case there was a clear temporal relationship to Natalizumab, further supported by a comprehensive investigation including laboratory, imaging, and biopsy. This case report highlights the rare, but important complication of DILI as a result of Natalizumab, in a patient who historically had shown good tolerance to it. Clinicians should be advised to monitor for hepatotoxicity in patients taking Natalizumab, especially in those who are retreated with the drug.

## Data Availability

Data were adapted from patient's clinical hospital course. Underlying data can be obtained, if needed, from patient's hospital chart, with approval from Northwestern Memorial Hospital eIRB.
